# Rising C-section use and the private maternal health care sector in Pakistan: evidence from the 2012–13 and 2017–18 Demographic and Health Surveys

**DOI:** 10.1186/s12884-025-08014-z

**Published:** 2025-10-27

**Authors:** Cynthia M. Cready, Saman Nazir, Tanya Faglie, Ronald Kwon

**Affiliations:** 1 Department of Sociology, University of North Texas, 1155 Union Circle Drive #311157, Denton, TX 76203-5017 USA; 2 Pakistan Institute of Development Economics, Quaid-i-Azam University Campus, P.O. Box 1091, Islamabad, 44000 Pakistan

**Keywords:** Cesarean section, Childbirth trends, Place of delivery, Mode of delivery, Health services use, Private providers, Medicalization, Heckman probit, Pakistan

## Abstract

**Background:**

Pakistan is part of a global escalation in cesarean section (C-section) rates, raising concerns that medically unnecessary procedures are being performed—particularly within the private maternal health care sector. Some studies have shown that C-section rates are higher in private than in public health facilities, possibly due to timesaving and profit-driven incentives among private-sector physicians. Consequently, Pakistan’s private health facilities may be more likely to perform nonessential C-sections than its public facilities. Building on these concerns, this paper examines how delivery at a private versus public facility in Pakistan affects the likelihood of a C-section, and how this likelihood has evolved over time.

**Methods:**

We used data from the 2012–13 and 2017–18 Pakistan Demographic and Health Surveys. Unlike previous studies, we employed a conceptual framework to identify predictors and used a Heckman probit model to adjust for selection bias—recognizing that access to facilities offering C-sections varies across sociodemographic groups. The model conceptualized childbirth as a two-step process: determining, first, the place of delivery (home versus facility), and second, the mode of delivery (vaginal versus C-section).

**Results:**

Accounting for selection bias and controlling for medical factors, women who delivered at private facilities were more likely to have a C-section. This likelihood increased from 3.6 to 14.2%-points between surveys. The larger gap in 2017–18 reflected an increase in the probability of a C-section in private facilities and a decrease in the probability of one in public facilities.

**Conclusions:**

These findings suggest that C-sections are increasingly performed for nonmedical reasons, especially in private facilities where financial incentives and weak regulation may influence decisions. Considering community misconceptions and varied attitudes toward childbirth, culturally sensitive public education is needed. Targeted workshops and media campaigns can provide women and families with clear information on the risks and long-term consequences of unnecessary C-sections.

**Supplementary Information:**

The online version contains supplementary material available at 10.1186/s12884-025-08014-z.

## Background

Cesarean section (C-section) is a surgical procedure in which a baby is delivered through incisions of the mother’s abdomen and uterus. Common medical indications for the procedure include previous C-section, cephalopelvic disproportion, fetal distress, and breech or other abnormal presentation [1–5]. When medically indicated, the procedure can be lifesaving [2, 3, 5]. However, when C-sections are performed without clear medical reasons, mothers and babies may be exposed to unnecessary risks that may outweigh the benefits. Compared to spontaneous vaginal delivery, C-sections performed without medical indications are associated with increased short-term risks of maternal death, admission to the intensive care unit, blood transfusion, hysterectomy, and severe perinatal outcomes, as well as long-term risks, including maternal complications in future pregnancies and childhood asthma and obesity [5–8].

The World Health Organization (WHO) maintains that C-sections "should ideally only be undertaken when medically necessary" and that every woman who needs one should have access to it ([9] p4). Although the WHO does not currently endorse a specific population-level C-section rate, it notes that rates higher than 10% are not associated with reduced maternal or neonatal mortality [9–11]. Guidelines for low- and middle-income countries (LMICs) developed by experts with the WHO’s support suggest that rates below 5% may reflect underuse, while rates above 15% may reflect overuse ([12] p39).

The global C-section rate is well above 15% and increasing [9–13]. From 1990 to 2018, it increased by 19%-points to 21% and is projected to reach 29% by 2030 [13]. Despite C-section rates for some low-income countries below 5% in 2018, rates increased in all country income groups from 1990 to 2018 [13]. They increased most rapidly in middle-income countries and are projected to average 37% in both middle- and high-income countries by 2030 [13]. The largest increases occurred in Eastern and Western Asia, where at least half of all births are expected to be delivered via C-section by 2030 [13]

Reasons for rising C-sections worldwide include medical factors, such as increased maternal age and obesity [14], as well as nonmedical factors, such as cesarean delivery on maternal request (CDMR) and various health care system arrangements [15, 16]. However, the extent to which these factors contribute to the increase in C-section use is not particularly well documented and may vary by country. For example, a 2011 systematic review and meta-analysis of 38 studies conducted across various countries found that only 16% of women expressed a preference for C-sections [17]. Interestingly, this preference was higher among women in middle-income countries compared to those in high-income countries (22% versus 12%). Additionally, a 2004–2008 survey of health facility medical records showed that the percentage of C-sections without medical indications was low—2% or less—in 23 of 24 countries, with China as the exception at 12% [5, 6].

Nonetheless, studies suggest that C-sections for nonmedical reasons have increased in China and other middle-income countries, potentially due to health system arrangements that facilitate CDMR or incentivize C-sections for physicians’ convenience, protection, or economic gain [1, 4, 15, 16, 18–27]. In China, attention has centered on varying insurance and payment schemes [22, 25]. In other middle-income countries, such as Pakistan and several of its regional neighbors, research has emphasized the higher rate of C-sections in private compared to public facilities and its possible links to timesaving and profit-driven incentives among private-sector physicians [15, 16, 18, 20, 21, 23, 24, 26, 27]. However, findings have been mixed [16, 18, 21, 26–31]. For example, analyses of the 1990–91, 2006–07, and 2012–13 Pakistan Demographic and Health Surveys (PDHSs) found no statistically significant difference in sector-specific rates after adjusting for sociodemographic and medical factors [28, 30], whereas 2017–18 PDHS data showed higher C-section use among women delivering in private facilities [18].

Although valuable, past studies have often relied on statistical criteria to select predictors and overlooked the two-step nature of facility-based C-section delivery, introducing selection bias. First, medical and sociodemographic characteristics influence the place of delivery (home versus health facility) [32–38]. Second, among facility deliveries, the mode of delivery (vaginal versus C-section) should ideally reflect medical need. Bias is heightened in out-of-pocket health care settings, where higher socioeconomic status (SES) increases access to facilities [32–35, 37]—and thus C-sections. In contrast, financially constrained women may only seek facility care when anticipating surgery. Analyses that ignore this nonrandom selection risk misinterpretation.

Pakistan presents a useful case study to more closely examine the relationship between rising C-section use and the formal private maternal health care sector. Despite persistent negative perceptions of C-sections among women and families in parts of the country [23, 39], its C-section rate tripled over a decade to 22% in 2017–18 [40–42], raising concerns about medically unnecessary procedures [9, 10]. These concerns are especially acute in LMICs like Pakistan, where fertility remains high (3.6 births per woman in 2017–18 [42]), health care is largely paid out of pocket, and health system capacity is strained [43–45].

Pakistan’s health care system is highly privatized, with 63% of health expenditures by nongovernment sources—91% directly from households ([46] pxiii). Maternal health care follows this pattern [47]. Only since 2020 has Pakistan begun expanding state-sponsored insurance for maternal health care services [48–50].

In 2017–18, most births in Pakistan occurred in the informal or formal private maternal health care sectors: 34% at home, typically by a traditional birth attendant or community midwife, and 44% in private health facilities [42, 47]. Only 22% were delivered in public facilities. Public sector services range from community outreach and basic preventive and curative care to tertiary hospitals with specialized and referral services, which are mainly located in urban centers [47]. Formal private sector services also vary and include small and medium-sized hospitals, maternal care centers, and "government-employed physicians and paramedics running clinics after office hours"( [23, 47] p15). Unlike public sector services, private health care in Pakistan is largely fee-for-service [23, 47]. In addition, a 2017–8 review of hospitals in the Khyber Pakhtunkhwa (KP) province found that private facilities use profit-sharing salary models and remain "mostly unregulated" ( [23, 44, 47] pp9-10,42). Moreover, although there is a systemwide need for quality assurance mechanisms, such as standard operating procedures and clinical guidelines, it is greater in the private sector ( [44] pp9-10). Consequently, private-sector physicians may be more incentivized to perform C-sections for convenience, financial gain, or at the request of women—particularly those able to afford higher costs—to avoid vaginal delivery or for other nonmedical reasons [23, 39, 51–55]. 

Given this background, the current study focuses on Pakistan’s private–public sector gap in C-section use. That is, controlling for other factors, it addresses how delivery at a private rather than a public health facility affects the likelihood of a C-section and how this likelihood has evolved over time. We extend earlier studies in three meaningful ways. First, since high C-section rates in Pakistan are a relatively recent phenomenon, we use the most recent waves of the PDHS (i.e., 2012–13 and 2017–18), which have become key sources of data for C-section growth in Pakistan [18, 28, 30]. Additionally, we pool the data to test for change between survey years. Importantly, we use data collected before widespread implementation and access to state-sponsored insurance for delivery services in Pakistan. Second, we account for selection bias by modeling childbirth more appropriately as a two-step process rather than considering the place of delivery (home versus health facility) and mode of delivery (vaginal versus C-section) separately. Third, we apply Andersen’s [56] Behavioral Model of Health Services Use to place and mode of delivery, making this one of the very few studies to adapt Andersen’s model to examine childbirth [57], let alone C-section procedures. In doing so, we use a conceptual framework to guide our analysis, rather than relying on statistical criteria to select predictors (except see [21, 27, 29]).

## Methods

### Data source and sample

We conducted secondary data analysis using a pooled cross-sectional study design. Data were drawn from the 2012–13 and 2017–18 Pakistan Demographic and Health Surveys (PDHSs) [41, 42]. The PDHS uses a stratified two-stage sample design to collect nationally and regionally representative information on demographic and maternal and child health indicators at the time of the survey. In the first stage, primary sampling units (PSUs), or "clusters" of households, were selected by the PDHS from each stratum (i.e., region, separated into urban and rural areas) using a list of enumeration blocks/villages identified by the Pakistan Bureau of Statistics. The second stage of sampling involved selecting households from each PSU. All ever-married women aged 15–49 in the selected households were eligible for interview. In 2012–13, 13,558 (93%) of eligible women were interviewed. The corresponding number for 2017–18 was 15,068 (95%). For the current study, we pooled the data from the two surveys. By increasing sample size, pooling enhanced the reliability of our estimates [58]. It also allowed us to examine the effect of type of health facility (private versus public) on C-sections over time by including time (i.e., survey year) as a variable in the analysis [59]. Pooling the data limited our analytical sample to respondents from five of the six regions that were included in both surveys. These five regions were Punjab, Sindh, Khyber Pakhtunkhwa (KP), Balochistan, and Islamabad and, according to Pakistan’s census, represented 98% of the country’s population of 207.6 million in 2017 [60]. Although Gilgit Baltistan (GB), an administrative territory of Pakistan, was also included in both surveys, it was excluded per the PDHS from the pooled dataset because it was weighted separately from the country’s other regions in 2017–18 [42].

These conditions reduced the sample to 12,342 women from the 2012–13 PDHS and 11,352 women from the 2017–18 PDHS for a combined total of 23,694 women from the two surveys. Of these women, 10,791 did not give birth in the 5 years prior to the survey, leaving 12,903, or 54%, who gave birth at least once in the five years prior to the survey. In cases of multiple births during the period, we analyzed the most recent one since the PDHS only asked about antenatal care for the most recent birth.

Overall, there were relatively few cases with missing values. Four variables accounted for 90% of the 231 cases with missing information: 37 cases were missing on one of the two dependent variables, and an additional 125 and 45 were missing on husband/partner’s education and number of antenatal visits, respectively. After listwise deletion, the final pooled sample for analysis consisted of 12,672 ever-married women aged 15–49 who gave birth in the five years prior to the survey and were not missing on any of the study variables. For a summary of the steps taken to construct the pooled dataset, see Table 1 in Supplementary Material 1.

### Analytic strategy

We used the Heckman probit model to account for potential selection bias [61, 62]. The model simultaneously estimates two equations—a place of delivery (selection) equation and a mode of delivery (outcome) equation—with correlated error terms. This correlation of error terms, rho (ρ), if different from 0, corrects the mode of delivery equation for potential differences between the health facility sample and the broader sample. Our study meets the criteria outlined by Morrissey et al. ( [63] p48) for the use of the model, including ensuring its identification by appropriately including SES indicators in the place of delivery (selection) equation and excluding them from the mode of delivery (outcome) equation. Although facility-based (versus home) delivery is affected by both SES and clinical/medical factors [32–38], C-section (versus vaginal) delivery, as a surgical procedure, is done in clinical/medical settings. Only women who deliver at a health facility are at "risk" of a C-section, which, again, is ideally only performed when medically indicated.

Analyses were conducted on the combined 2012–13 and 2017–18 dataset using Stata 16 (StataCorp, College Station, Texas, USA) and included weights to account for the sampling design.

### Dependent variables

We constructed the dependent variable for the selection equation based on women’s responses to the question regarding their most recent birth: "Where did you give birth to (NAME)?" Births in a health facility, regardless of sector, were coded 1, and births at home were coded 0. The dependent variable for the outcome equation on facility-based births was derived from responses to the question: "Was (NAME) delivered by caesarean, that is, did they cut your belly open to take the baby out?" Responses indicating C-section delivery ("yes") were coded 1, while vaginal deliveries ("no") were coded 0.

### Conceptual framework and independent variables

**Fig. 1:**
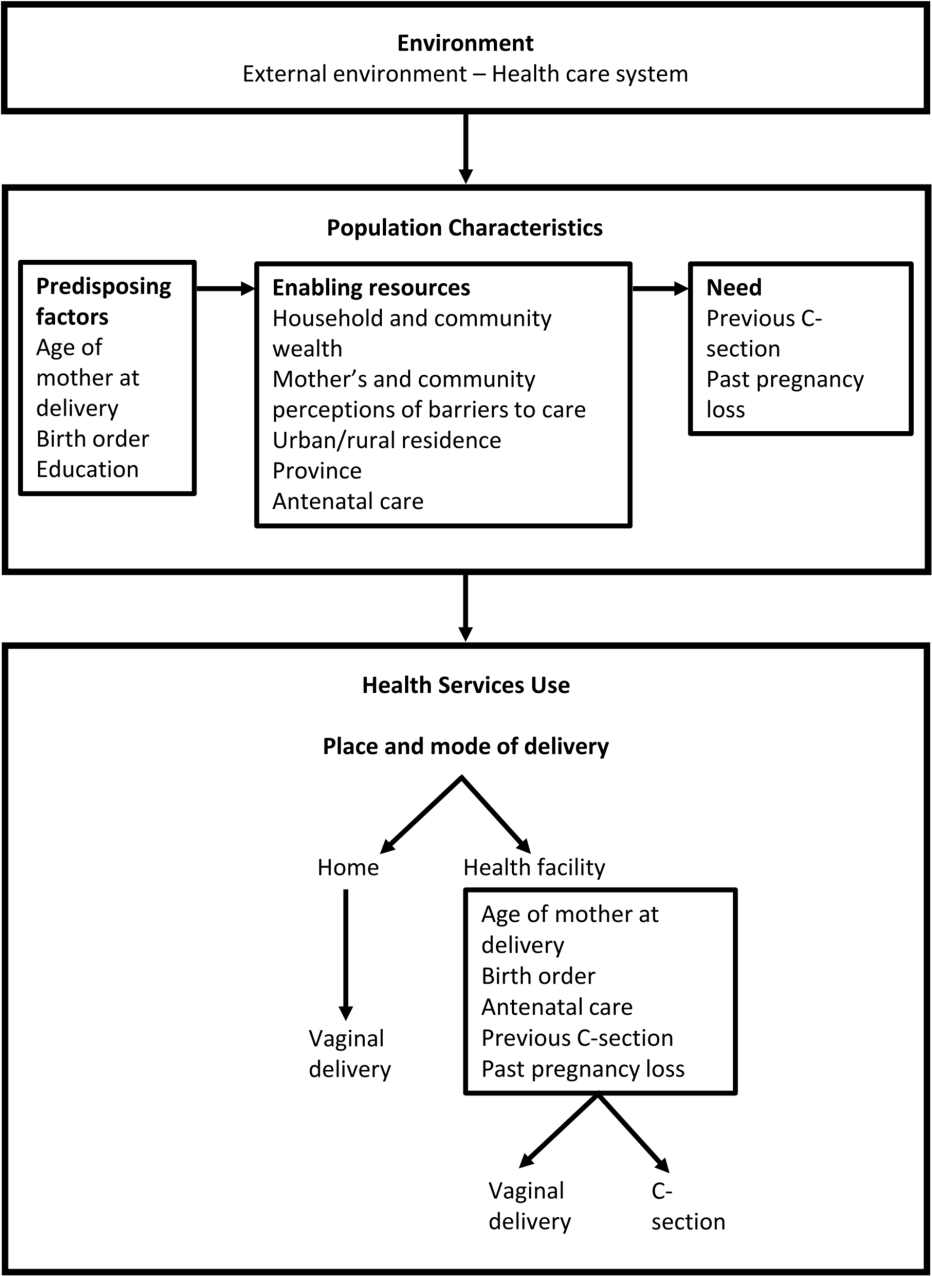
Place and mode of delivery and their determinants. Adapted from Andersen’s [56] Behavioral Model of Health Services Use

The selection of independent variables was guided by Andersen's Behavioral Model of Health Services Use [56] and informed by previous studies in Pakistan and other LMICs [18, 28–38]. The selection was also limited to information collected by the PDHS in both survey years. As our adaptation of Andersen’s model in Fig. 1 shows, place and mode of delivery are determined by environment and population characteristics. Environment includes the legal and health care system [64, 65]. Population characteristics can be separated into predisposing factors, enabling resources, and need [66].

#### Predisposing factors for health facility (versus home) delivery

In the current study, predisposing factors for health facility versus home delivery included birth order, mother's age at delivery, and education. Birth order of the respondent's most recent birth in the five years prior to survey was represented by three binary variables, coded 1 for second-, third-, and fourth- or higher-order births, respectively, with first-order births serving as the reference [67]. We calculated the age of women at their most recent birth and recoded it as two binary variables coded 1 for "20–29 years" and "30 + years," respectively, with age "< 20 years" serving as the reference. We based this categorization on age-associated risks for pregnancy and delivery complications [68]. Following previous studies [57, 66], we included both the mother's and the husband's/partner's education. For each, we used three binary variables to represent the primary, secondary, and higher levels, respectively, with "no formal education" as the reference category.

#### Enabling resources for health facility (versus home) delivery

According to Andersen [56], predispositions for using health care services are not enough—enabling resources, the second element of population in the model, must be present. In the current study, enabling resources included wealth, women's empowerment in health-seeking behaviors, province, residential status, and antenatal care.

Wealth was measured at both the household and community levels. At the household level, we used the wealth index constructed by the PDHS from information collected from interviewees on various household amenities, such as flooring, source of water, availability of electricity, and possession of durable consumer goods, to name a few. The PDHS coded the index in the dataset as quintiles (0 = poorest, 1 = poor, 2 = middle, 3 = rich, and 4 = richest) [41, 42]**.** For the analysis, we coded four binary variables 1 for each of the top four quintiles, respectively, with the poorest quintile serving as the reference. At the community level, we calculated the average household wealth quintile (coded from 0 to 4) for all women interviewed by the PDHS in the women's "community"or PSU (whether they were in our sample or not) [69].

Empowerment in health-seeking behaviors was measured using women’s responses to questions asking if getting permission to see the doctor, money needed for treatment, or distance to the health facility were big problems in getting medical help for themselves [70]. Specifically, we constructed a variable coded 1 for each potential barrier if it was *not* a big problem and 0 otherwise. For a measure at the community level, we counted the number of potential barriers that were *not* a big problem for each woman interviewed by the PDHS in the respondent's PSU and took the average.

Residential status was coded 1 for living in an urban area and 0 otherwise. For province, we coded four binary variables 1 for Sindh, KP, Balochistan, and Islamabad (capital city), respectively, with Punjab serving as the reference.

Lastly, although antenatal care technically falls under health care services use in Andersen’s model [56, 66], we included the variable among enabling resources for facility-based versus home delivery since having a usual source of care tends to facilitate additional contact within a health care system [33, 57]. The WHO recommendation regarding the minimum number of antenatal care visits changed from four to eight between the 2012–13 and 2017–18 administrations of the PDHS ( [71] px). Since we pooled the data from the two surveys, we categorized antenatal visits based on the earlier WHO recommended minimum of four visits. Specifically, we coded two binary variables, 1 for "1–4 visits" and "5 + visits," respectively, with "no visits" as the reference category.

#### Need for health facility (versus home) delivery

Need, the third element of population in Andersen’s model [56], is influenced by the previously discussed predisposing and enabling factors. Need can be perceived or evaluated. Either way, both pregnancy loss and having a C-section may put the mother at risk of facility-based delivery via C-section for subsequent births [1, 4, 16, 29]. Thus, in the current study, we included past pregnancy loss and previous C-sections as indicators of need for facility-based delivery. The PDHS constructed past pregnancy loss from women's responses to survey questions about their reproductive history. They coded the variable 1 for ever having a pregnancy that did not result in a live birth (i.e., a miscarriage, abortion, or stillbirth) and coded it 0 otherwise. We used information on women's births in the five years prior to the survey, other than the most recent one, to construct an indicator of previous C-section delivery, coded 1 for yes and 0 otherwise.

#### Indicators for C-section (versus vaginal) delivery

As shown in the bottom panel of Fig. 1, five clinical/medical indicators (variously representing "predisposing factors," "enabling factors," and "need" in the middle panel predicting place of delivery) were also included as predictors in the mode of delivery equation: age at delivery, birth order, antenatal care, previous C-section, and past pregnancy loss. We also included survey year, type of health facility, and the interaction between these variables in the mode of delivery equation. These variables addressed whether, all else equal, women who delivered at a private facility were more likely to have a C-section than those who delivered at a public facility and, if so, whether this likelihood changed over time. Survey year was coded 1 for 2017–18 and 0 for 2012–13. Facility-based deliveries were coded 1 for private and 0 for public. The interaction between survey year and type of facility was represented by their product.

## Results

### Univariate and bivariate analysis

Table 1 presents descriptive statistics for variables used in the analysis. The first column of data in the table shows the distribution of each variable used in the place of delivery equation for all births in the sample (*n* = 12,672). The second column of data in the table shows the distribution of each variable used in the mode of delivery equation for the facility-based births in the sample (*n* = 7553). About 60% of all births in the sample occurred at a health facility, and about 33% of those occurring at a facility were delivered via C-section.

**Table 1:** Descriptive Statistics for Variables Used in the Analysis of the Pooled 2012–13 and 2017–18 PDHSs

Variable	% of All Births	% of Health Facility Births
	**(*n* = 12,672)**	**(*n* = 7553)**
**Place of Delivery**		
Home	40.1	
Health facility	59.9	
**Mode of Delivery**		
Vaginal		66.9
C-section		33.1
**Clinical/Medical Indications**		
Previous C-section		
No	92.6	87.9
Yes	7.5	12.1
Past pregnancy loss		
No	66.8	66.9
Yes	33.2	33.1
Age at delivery		
< 20	7.3	7.1
20–29	57.6	60.2
30 +	35.1	32.7
Birth order		
1st	19.5	24.3
2nd	20.4	23.4
3rd	17.0	17.9
4th +	43.2	34.4
Antenatal visits		
0	18.4	6.0
1–4	49.2	47.2
5 +	32.4	46.8
**Socioeconomic Status**		
Mother's formal education		
None	51.6	
Primary	16.6	
Secondary	20.5	
Higher	11.3	
Husband's/partner's formal education		
None	31.0	
Primary	16.4	
Secondary	34.7	
Higher	17.9	
Household wealth index quintile		
Poorest	21.8	
Poorer	19.9	
Middle	20.3	
Richer	20.1	
Richest	18.0	
**Mother's Perceptions of Barriers to Medical Care**		
Getting permission to see doctor		
Big problem	21.7	
Not a big problem	78.3	
Getting money for advice/treatment		
Big problem	32.0	
Not a big problem	68.0	
Distance to health facility		
Big problem	41.9	
Not a big problem	58.1	
**Community Characteristics**		
Residential status		
Rural	68.0	
Urban	32.0	
Province		
Punjab	54.7	
Sindh	23.7	
Khyber Pakhtunkhwa	15.9	
Balochistan	5.2	
Islamabad	0.6	
Average woman's perception of barriers to medical care		
< = 2.08 (of 3) potential barriers not a big problem	40.3	
> 2.08 (of 3) potential barriers not a big problem	59.7	
Average household wealth index quintile		
< = 1.95	52.1	
> 1.95	47.9	
**Type of Health Facility**		
Public		32.0
Private		68.0
**Survey Year**		
2012–13	53.4	46.1
2017–18	46.6	53.9

*PDHS* Pakistan Demographic and Health Survey. Percentages for each variable may not total 100.0 due to rounding

The bivariate analysis results in Table 2 show that facility-based delivery increased from 52% in 2012–13 to 69% in 2017–18 (*p* < 0.001). Women with higher SES—such as greater education and household wealth—and fewer reported barriers to medical care were significantly more likely to have facility-based births (*p* < 0.001). Only 21% and 7% of women with secondary or higher education, respectively, delivered at home compared to 55% with no formal education. Women whose husbands/partners had secondary or higher education and those whose households were in the top wealth quintile were also less likely to deliver at home. Furthermore, nearly two-thirds (63%−66%) of women who indicated that permission, money, or distance was "not a big problem" had a facility-based birth compared to only about one-half (46%−51%) of women who indicated that these factors were "a big problem."

**Table 2:** Bivariate Statistics for Variables Used in the Analysis of the Pooled 2012–13 and 2017–18 PDHSs

**Variable**	**Place of Delivery**		**Mode of Delivery**	
	**(*n* = 12,672)**		**(*n* = 7553)**	
	**% Home**	**% Facility**	**% Vaginal**	**% C-section**
**Clinical/Medical Indications**				
Previous C-section				
No	43.1	56.9***	74.8	25.2***
Yes	3.0	97.0	9.1	90.9
Past pregnancy loss				
No	40.1	59.9	66.9	33.1
Yes	40.2	59.9	67.0	33.0
Age at delivery				
< 20	41.2	58.8***	77.6	22.5***
20–29	37.5	62.5	65.4	34.6
30 +	44.2	55.8	67.3	32.7
Birth order				
1st	25.3	74.7***	60.9	39.1***
2nd	31.5	68.5	63.1	36.9
3rd	36.6	63.4	63.1	36.9
4th +	52.3	47.8	75.7	24.4
Antenatal visits				
0	80.5	19.5***	87.7	12.3***
1–4	42.6	57.5	74.2	25.8
5 +	13.5	86.5	56.9	43.1
**Socioeconomic Status**				
Mother's formal education				
None	55.4	44.6***		
Primary	38.1	61.9		
Secondary	21.5	78.5		
Higher	7.2	92.8		
Husband's/partner's formal education				
None	56.5	43.5***		
Primary	46.6	53.4		
Secondary	33.4	66.6		
Higher	18.8	81.2		
Household wealth index quintile				
Poorest	62.0	38.0***		
Poorer	55.4	44.6		
Middle	41.3	58.8		
Richer	26.0	74.0		
Richest	11.2	88.8		
**Mother's Perceptions of Barriers to Medical Care**				
Getting permission to see doctor				
Big problem	51.1	48.9***		
Not a big problem	37.1	62.9		
Getting money for advice/treatment				
Big problem	53.8	46.2***		
Not a big problem	33.7	66.3		
Distance to health facility				
Big problem	48.6	51.4***		
Not a big problem	34.0	66.0		
**Community Characteristics**				
Residential status				
Rural	47.9	52.1***		
Urban	23.4	76.6		
Province				
Punjab	39.0	61.0***		
Sindh	31.1	68.9		
Khyber Pakhtunkhwa	47.6	52.4		
Balochistan	73.3	26.7		
Islamabad	13.2	86.9		
AVG woman's perception of barriers to medical care				
< = 2.08 (of 3) potential barriers not a big problem	52.5	47.5***		
> 2.08 (of 3) potential barriers not a big problem	31.8	68.2		
AVG household wealth index quintile				
< = 1.95	53.0	47.0***		
> 1.95	26.1	73.9		
**Type of Health Facility**				
Public			73.3	26.7***
Private			63.9	36.1
**Survey Year**				
2012–13	48.4	51.6***	69.3	30.7*
2017–18	30.6	69.4	64.9	35.1

*PDHS* Pakistan Demographic and Health Survey, *AVG* average. Row percentages for each dependent variable may not total 100.0 due to rounding. *p ≤.05,***p ≤.001 (design-based *F* tests)

Urban women were significantly more likely to deliver at a health facility compared to rural women (*p* < 0.001). Additionally, community factors—such as local wealth and fewer barriers to care—were associated with an increase in facility-based deliveries (*p* < 0.001). Women in the capital city of Islamabad were most likely (87%) to deliver at a facility, whereas women from the more rural Balochistan province were least likely (27%) to do so (*p* < 0.001).

As for clinical/medical factors, a previous C-section, giving birth in one's twenties, a lower-order birth, and antenatal care were significantly associated with facility-based delivery (*p* < 0.001), whereas a history of miscarriage, abortion, or stillbirth was not.

Like the facility-based delivery rate, the C-section rate in Pakistan also increased over time, from 31% in 2012–13 to 35% in 2017–18 (*p* < 0.05). Although past pregnancy loss was not significantly related to the mode of delivery in health facilities, all other clinical/medical factors were (*p* < 0.001). An overwhelming majority (91%) of women who previously had a C-section in the five years prior to the survey delivered their most recent baby via C-section compared to only one-fourth (25%) of women who had not previously had a C-section. Women in their twenties were more likely (35%) to deliver via C-section than adolescent girls (23%) or women in their thirties or older (33%). C-sections were less likely at higher birth orders (24% versus 37%−39%) and among women without antenatal care (12% versus 26%−43%). Finally, C-sections were more prevalent in private health facilities (*p* < 0.05). About 36% of women who delivered in a private facility had a C-section compared to 27% of women who delivered in a public facility.

### Multivariable analysis

Tables 3 and 4 present the coefficients from the Heckman probit model, which adjusts for selection bias by jointly estimating the factors influencing both the place and mode of delivery. Two separate models were estimated. The first includes all main effects. The second (not shown) adds an interaction with time. Both models were statistically significant (*p* < 0.001), and estimates of rho, the correlation between the error terms of the selection and outcome equations in each model, were significantly different from 0 at the 0.001 level, indicating sample selection bias.

**Table 3:** Results of Heckman Probit Regression: Step 1, Selection, Place of Delivery (*n* = 12,672)

**Variable**		**Health Facility (versus home)**	
		**Coefficient**	**Linearized SE**
**Clinical/Medical Indications**			
Previous C-section (no)	Yes	1.504***	0.128
Past pregnancy loss (no)	Yes	0.071*	0.035
Age at delivery (< 20)	20–29	0.155*	0.068
	30 +	0.270***	0.076
Birth order (1st)	2nd	−0.300***	0.060
	3rd	−0.398***	0.062
	4th +	−0.604***	0.062
Antenatal visits (0)	1–4	0.728***	0.054
	5 +	1.270***	0.067
**Socioeconomic Status**			
Mother's formal education (none)	Primary	0.025	0.053
	Secondary	0.145*	0.060
	Higher	0.477***	0.085
Husband's/partner's formal education (none)	Primary	−0.042	0.053
	Secondary	0.081 +	0.044
	Higher	0.175**	0.057
Household wealth index quintile (poorest)	Poorer	0.044	0.061
	Middle	0.166*	0.069
	Richer	0.356***	0.079
	Richest	0.541***	0.105
**Mother's Perceptions of Barriers to Medical Care**			
Permission (big problem)	Not a big problem	−0.045	0.058
Money (big problem)	Not a big problem	0.089 +	0.047
Distance (big problem)	Not a big problem	−0.033	0.045
**Community Characteristics**			
Residential status (rural)	Urban	−0.103	0.078
AVG household wealth index quintile		0.083 +	0.049
AVG woman's perceptions of barriers to medical care		0.035	0.063
Province (Punjab)	Sindh	0.437***	0.086
	KP	0.070	0.085
	Balochistan	−0.353***	0.109
	Islamabad	0.199*	0.093
**Survey year** (2012–13)	2017–18	0.365***	0.056
Constant		−1.125***	0.165

*SE* standard error. Reference categories are in parentheses. + *p* ≤.10, *p ≤.05 , **p ≤.01, ***p ≤.001 (two-tailed tests)

**Table 4:** Results of Heckman Probit Regression: Step 2, Outcome, Mode of Delivery (*n* = 7553)

**Variable**		**C-section (versus vaginal)**	
		**Coefficient**	**Linearized SE**
**Clinical/Medical Indications**			
Previous C-section (no)	Yes	1.977***	0.107
Past pregnancy loss (no)	Yes	0.091 +	0.051
Age at delivery (< 20)	20–29	0.383***	0.089
	30 +	0.647***	0.107
Birth order (1st)	2nd	−0.541***	0.070
	3rd	−0.482***	0.082
	4th +	−0.682***	0.091
Antenatal visits (0)	1–4	0.008	0.141
	5 +	0.218	0.182
**Type of Health Facility** (public)	Private	0.301***	0.048
**Survey Year** (2012–13)	2017–18	0.040	0.052
Constant		−0.831***	0.226
Rho		−0.469***	
Design F(11, 850)		43.44***	

*SE* standard error. Reference categories are in parentheses. + *p* ≤.10, ***p ≤.001 (two-tailed tests)

Focusing first on place of delivery in Table 3, in 2017–18, women were significantly more likely to deliver at a health facility compared to 2012–13 (*p* < 0.001). Higher educational levels for both mothers and their husbands/partners, as well as household wealth, were positively associated with facility-based delivery (*p* < 0.05). Moreover, controlling for the effects of these objective SES indicators, women who perceived that getting money for medical advice/treatment was not a big problem were more likely to have a facility-based birth (*p* < 0.10).

At the community level, facility-based delivery was more likely among women living in wealthier areas, regardless of their personal or household socioeconomic resources (*p* < 0.10). Place of delivery also varied by province. Compared to women from Punjab, women from Sindh and Islamabad were more likely to deliver at a health facility than at home, whereas women from Balochistan were less likely to do so (*p* < 0.05). Although the bivariate analysis showed that urban women and those living in a community where the average woman reported few barriers to medical care were more likely to deliver at a facility, these associations did not hold up in the multivariable analysis.

All five clinically related factors were statistically significant (*p* < 0.05). Facility-based delivery was associated with previous C-sections, past pregnancy loss, and more antenatal care visits. Adolescent girls were less likely than older women to deliver at a facility. However, all else equal, first order births were more likely than higher order births to be delivered there.

Table 4 presents estimates for the mode of delivery equation. Although neither year nor antenatal care was significantly related to the mode of delivery, the other predictors in the equation were. Women with a history of C-section delivery (*p* < 0.001) or pregnancy loss (*p* < 0.10) were more likely to deliver their most recent baby via C-section than vaginally. C-section delivery was also more likely among older women (*p* < 0.001), but it was less likely among women with higher-order births (*p* < 0.001). After controlling for clinical factors, deliveries at private facilities were significantly more likely to be via C-section compared to those at public facilities (*p* < 0.001). These results are consistent with the hypothesis that nonmedical factors may contribute to higher C-section rates in private health care settings.

**Fig. 2:**
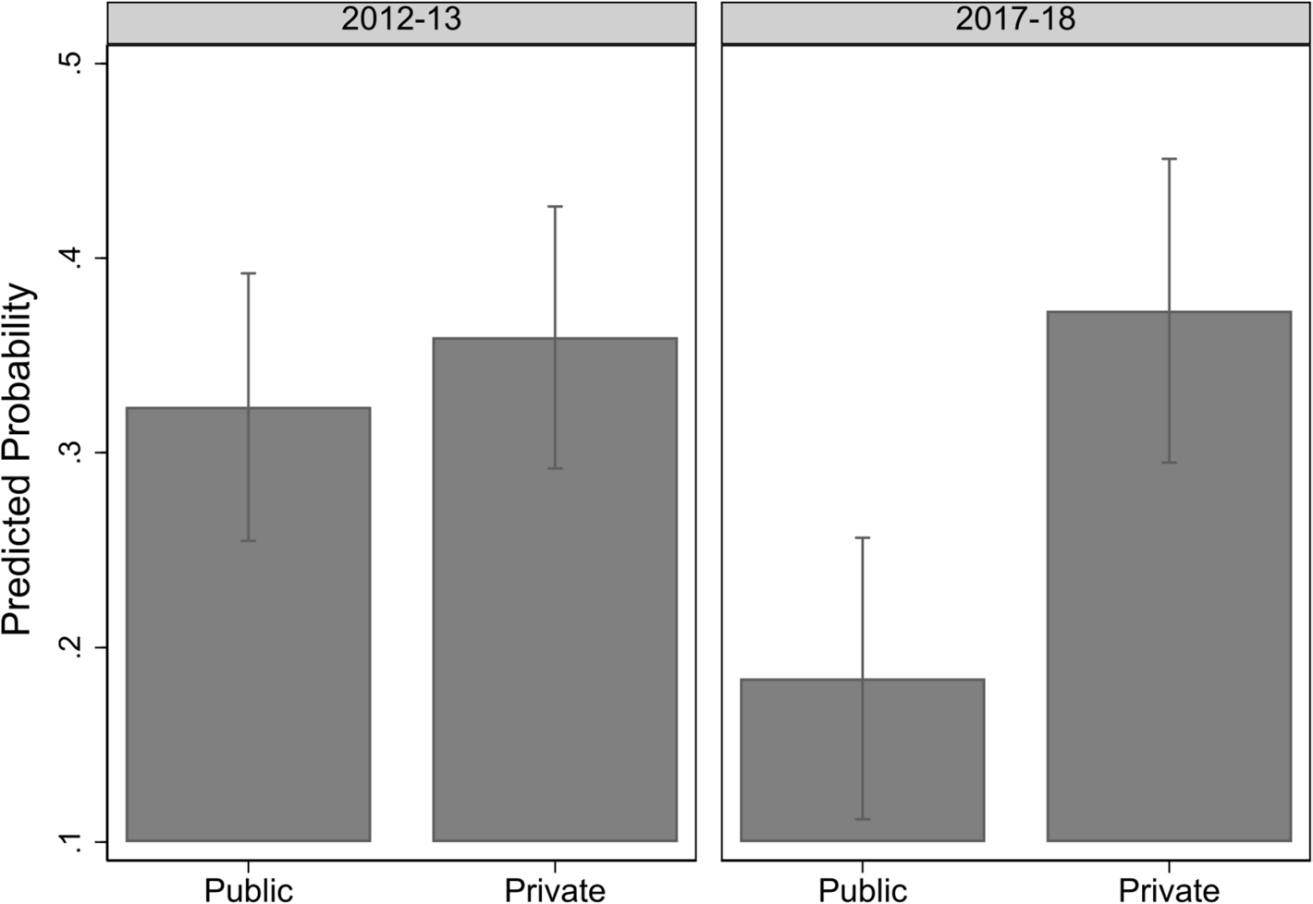
Effect of type of health facility on predicted probability of C-section delivery over time

To examine whether the effect of the type of facility on C-sections differed over time, an interaction term (year*type) was added to the model. The likelihood of C-sections in private facilities significantly increased from 2012–13 to 2017–18 (*p* < 0.05). As predictive margins derived from the interaction model presented in Fig. 2 show [59], in 2012–13, private facility deliveries had a 3.6%-points higher probability of C-sections compared to public facilities (0.359 versus 0.324). However, this difference was not statistically significant (*p* = 0.097). By 2017–18, this gap had widened significantly to 14.2%-points (0.405 versus 0.263, *p* < 0.001), emphasizing the growing disparity between private and public facilities.

In sensitivity analyses, we estimated the model separately by survey year. We also estimated it excluding KP (where a limited, state-sponsored insurance pilot program was initiated in late 2015 [48–50]). Results were consistent with the pooled results presented and are included in Tables 2–5 and Fig. 1 in Supplementary Material 1.

## Discussion

Medically unnecessary C-sections are associated with increased risks, including maternal complications in future pregnancies and unnecessary neonatal interventions, which contribute to the over-medicalization of childbirth [5–8]. For some scholars, clinicians, and policymakers, this is a worrying trend, particularly for LMICs like Pakistan, whose C-section rates have risen in line with global trends [43, 45]. Due to the potential health risks and economic costs associated with medically unnecessary C-sections, concerns within the maternal and neonatal health community have intensified [9, 10, 13].

With its rising C-section rate, elevated fertility levels, and predominantly privatized health care system, Pakistan presents a unique and policy-relevant context for assessing the role of the formal private maternal health care sector in the provision of possibly unnecessary C-sections [16, 43, 45]. Some studies have shown that C-section rates are higher in private than in public health facilities, possibly due to timesaving and profit-driven incentives among private-sector physicians [15, 16, 18, 20, 21, 23, 24, 26, 27]. Consequently, Pakistan’s private health facilities may be more likely to perform nonessential C-sections than its public facilities [18, 23, 39, 44, 46, 47]. To explore this possibility further, we used pooled data from the 2012–13 and 2017–18 PDHSs to examine how delivery at a private rather than a public facility in Pakistan affects the likelihood of a C-section, and how this likelihood has evolved over time.

Previous research using these datasets has yielded mixed findings and suggests that elevated C-section rates in private facilities may be a relatively recent trend [18, 28, 30]. Unlike these studies, our analysis employed a conceptual framework to identify predictors and used a Heckman probit model to adjust for selection bias—recognizing that access to facilities offering C-sections varies across sociodemographic groups. The model conceptualized childbirth as a two-step process: determining, first, the place of delivery (home versus facility), and second, the mode of delivery (vaginal versus C-section).

Accounting for selection bias and controlling for medical factors, we found that women who delivered at private facilities were more likely to have a C-section. This likelihood increased from 3.6 to 14.2%-points over the five years between surveys. The widening gap in 2017–18 was driven by an increase in C-sections at private facilities and a decrease at public facilities. This trend may reflect resource constraints in the public sector or differing clinical guidelines that limit nonessential C-sections.

To better understand these patterns, it is important to consider Pakistan’s health system context. Pakistan’s spending on health is among the lowest in southern Asia [72, 73], while its population is one of the fastest growing in the region [72]. Although health spending increased slightly between the two surveys—from 0.6% of the gross domestic product in 2012–13 to 1.0% in 2017–18 [72]—this low level of investment, relative to a population growth rate of 2.0% per year [72], may have contributed to the lower probability of C-sections in the public sector in 2017–18 compared to 2012–13.

However, Pakistan’s public hospitals, particularly those providing tertiary care, often have more extensive infrastructure than hospitals in the private sector [44]. Consequently, a substantial number of deliveries in public hospitals are referrals, that is, failed home deliveries or "at risk" patients from private hospitals ( [44] p33). This patient mix might be expected to increase the probability of C-sections in the public sector and narrow the private–public gap, but we controlled for differences in patient mix across sectors with the inclusion of medical/clinical indications for C-sections in the model.

In contrast, the private sector operates under different financial and regulatory conditions that may encourage nonessential C-sections. Although the PDHS data did not allow us to examine the effects of differences in specific clinical guidelines and practice across sectors, a 2017–18 review of hospitals in the KP province noted differences in the payment structures and regulatory frameworks across sectors that may limit nonessential C-sections in public health facilities compared to private facilities [44]. Due to the "fee-for-service" and "profit-sharing" payment structures of private health facilities ( [44] p42), and the higher cost of C-section (versus vaginal) delivery, physicians in these facilities may be more incentivized to perform nonessential C-sections than those in public facilities. It may also be easier to perform these C-sections in the private sector. Compared to the public sector, the private sector is "mostly unregulated" and consists of "a range of providers from reputable hospitals to unregistered single-operator quacks" ( [44] pp9,19). Additionally, while there is a systemwide need for quality assurance mechanisms, such as "written standard clinical protocols and pathways for diagnostic and therapeutic procedures" and "monitoring, feedback, [and] review" protocols, the need is greater in the private sector ( [44] pp9-10,31).

Our analysis also identified several individual-level medical/clinical indications associated with cesarean delivery. Consistent with previous studies, we found that C-sections were more likely among women with older maternal age, lower birth order, a history of pregnancy loss, and a previous C-section [16]. Interestingly, as prior research suggests, citing a "previous C-section" as the reason for a subsequent C-section does not necessarily imply a clear medical indication ( [1] p8). Thus, the effect of a prior C-section—combined with Pakistan’s high fertility rate—suggests that initial procedures, whether medically necessary or not, may lead to subsequent C-sections that are medically unnecessary. These repeated interventions pose avoidable risks to maternal and neonatal health, with the danger increasing with each prior C-section [74].

### Recommendations

To address the rising trend of nonmedical C-sections, we suggest several interventions.

#### Interventions targeted at women and their families

For women and their families, we recommend implementing psychoeducational workshops/programs and media campaigns to empower them with knowledge of the risks and benefits of C-sections, including repeat C-sections [10, 15, 75]. These interventions could potentially reduce unnecessary procedures and improve maternal and neonatal health outcomes, especially if they are implemented with sensitivity to the negative perceptions of C-sections in some parts of Pakistan [23, 39]. Although it did not include any studies from Pakistan—and most were conducted in high-income countries—a recent systematic review of nonclinical interventions aimed at reducing medically unnecessary C-sections lends some support to these suggestions [76].

#### Interventions targeted at health care providers and systems

The same review also identified several successful interventions for health care providers and systems [76]. These interventions included "using clinical practice guidelines combined with mandatory second opinion for [C-section] indication" and "using clinical practice guidelines combined with audit and feedback about [C-section] practices" ( [76] p3). Regulatory frameworks should include evidence-based guidelines, mandatory second opinions for elective C-sections, and regular audits to monitor adherence, all aimed at curbing unnecessary procedures in both private and public facilities (e.g., [10, 15, 18]).

It is evident Pakistan needs such regulatory frameworks, especially with the recent introduction of limited state-sponsored insurance schemes to cover maternal health care services. The first such initiative in late 2015 was the Sehat Sahulat Program (SSP). This pilot program provided free health insurance to families living below the poverty line in a small number of districts in the KP province. Unfortunately, the lack of accountability in the formal private health care sector [44], combined with its profit-sharing and fee-for-service structure and the moonlighting of government physicians in its facilities, has increased tensions between it and the public sector under the SSP [44, 77]. For example, private hospitals’ larger share of revenue under the SSP may increase financial incentives for C-sections, potentially fueling higher rates of unnecessary C-sections and exacerbating the private–public gap in maternal care [78]. These tensions should be addressed.

To address them, the KP province’s government approved a "funds retention formula," whereby participating public hospitals in the province were to retain 75% of the income generated from treating SSP beneficiaries and to distribute 25% of this amount to the physicians who treated them [77]. Until recently, however, government physicians were not getting their share since the formula was largely unenforced until 2020 [78]. Additional efforts by the KP government to balance public–private sector participation in the SSP included reassessing private hospitals and restricting seven common surgeries—including C-sections—to public hospitals in 2023 [79, 80]. These policy changes may further strain the public sector infrastructure and, given the fee-for-service reimbursement structure of the SSP, they may also inadvertently incentivize public hospitals to perform unnecessary C-sections, as C-sections are more lucrative compared to vaginal deliveries. Considering all this, enhancing infrastructure and implementing control systems in both the public and private sectors are critical as Pakistan continues to move toward universal health coverage [48].

However, recent qualitative research in the U.S. has shown that implementing control systems involves a delicate balancing act with patient-centered care and may lead to unintended consequences. Morris concludes: "Hospital administrators, ACOG [American College of Obstetricians and Gynecologists], courts, malpractice insurers, and reinsurers have defined c-sections as the best practice *to protect themselves and maternity providers from blame in the case of a bad outcome*" (italics in original, ( [65] p153). Yet, Diamond-Brown found that malpractice threat was not mentioned as a factor in decision-making by half of the obstetricians interviewed, and that at least some were willing to break the "rules" on a case-by-case basis, justifying their choices "based on clinical experience or patient choice" ( [64] p53).

### Limitations

Our study has some limitations. Although the 2012–13 and 2017–18 administrations of the PDHS provide nationally representative, standardized data for comparing trends across five years, their cross-sectional design precludes causal inference. Additionally, the PDHS relies on maternal self-report, a data source that may be influenced by recall and social desirability biases, potentially affecting the validity of findings. The PDHS also does not provide near-birth labor complications history, which would be beneficial in accessing medical/clinical indications for C-sections more precisely [1, 4]. Nevertheless, it seems unlikely that these indications are differentially present by sector to explain the private–public sector gap in C-section use. Furthermore, PDHS data do not distinguish among types of private facilities, limiting insights into specific characteristics that may drive higher C-section rates. Differentiating between facility types could inform targeted policy recommendations to regulate practices in clinics versus hospitals. It could also shed light on the possibility that women needing or at greater risk of needing a medically necessary C-section are seeking out private hospitals for their deliveries because these hospitals offer higher quality services.

Lastly, mixed-methods studies, including those of health facilities and providers, would be fruitful in answering these questions and testing Andersen's [56] Behavioral Model of Health Services Use. Our use of the model was an improvement over not using any conceptual framework or relying solely on statistical criteria to select predictors. However, as Babitsch et al. [66] indicate, using secondary data alone is a limitation in fully testing the model.

## Conclusion

We examined rising C-section use in Pakistan and its potential link to the private maternal health care sector in Pakistan and found that women who delivered in private health facilities were more likely to have a C-section compared to those who delivered in public facilities and that this likelihood increased over time. These findings suggest that medically unnecessary C-sections are being performed, particularly in private facilities. Recommendations include the implementation of culturally sensitive psychoeducational workshops/programs and media campaigns to empower women and their families with knowledge of the risks and benefits of C-sections, and the development of regulatory frameworks that constrain nonessential C-sections in both the private and public health sectors. The study provides an important reference point for Pakistan and similar LMICs, especially those moving toward universal health coverage.

## Supplementary Information

Supplementary Material 1.

## Data Availability

The research used publicly available secondary data from two waves of the Pakistan Demographic and Health Survey (DHS) (available from The DHS Program funded by USAID at https://dhsprogram.com/).

## Abbreviations

CDMR: Cesarean delivery on maternal request
C-section: Cesarean section
GB: Gilgit Baltistan
KP: Khyber Pakhtunkhwa
LMICs: Low- and middle-income countries
PDHS: Pakistan Demographic and Health Survey
PSU: Primary sampling unit
SE: Standard error
SES: Socioeconomic status
SSP: Sehat Sahulat Program
WHO: World Health Organization
