# Supplementary material for: Rising C-section use and the private maternal health care sector in Pakistan: evidence from the 2012–13 and 2017–18 Demographic and Health Surveys
Source: BMC Pregnancy Childbirth. 2025 Oct 27;25:1137. doi: 10.1186/s12884-025-08014-z (PMC12560495; doi:10.1186/s12884-025-08014-z)
Supplement: Supplementary file 1 — Supplementary Material 1. [file 12884_2025_8014_MOESM1_ESM.pdf]

Supplementary Table 1
Determination of Final Pooled Sample

	Public-use Dataset		
	**2012-13 PDHS**	**2017-18 PDHS**	**Combined PDHS**
**<u>Initial Sample</u>**			
Ever-married women aged 15-49	13558	15068	28626
**<u>Sample Exclusion Criterion</u>**			
From AJK or FATA, as these regions were not surveyed in 2012-13	(0)	(2732)	(2732)
From GB, as there was a weighting issue for this region in 2017-18	(1216)	(984)	(2200)
Did not give birth in 5 years prior to survey	(5590)	(5201)	(10791)
Had a missing value on any variable used in the analysis	(79)	(152)	(231)
**<u>Final Sample</u>**	6673	5999	**12672**

Notes: PDHS=Pakistan Demographic and Health Survey. AJK=Azad Jammu and Kashmir. FATA=former Federally Administered Tribal Areas. GB=Gilgit Baltistan.

Supplementary Table 2
Results of Heckman Probit Regression: Step 1, Selection, Place of Delivery, 2012-2013 (*n*=6673)

Variable		Health Facility (versus home) Coefficient	Linearized SE
**Clinical/Medical Indications**			
Previous C-section (no)	Yes	1.544***	0.169
Past pregnancy loss (no)	Yes	0.121**	0.045
Age at delivery (<20)	20-29	0.242*	0.097
	30+	0.371***	0.111
Birth order (1st)	2nd	-0.283***	0.080
	3rd	-0.486***	0.087
	4th+	-0.664***	0.085
Antenatal visits (0)	1-4	0.736***	0.065
	5+	1.234***	0.088
**Socioeconomic Status**			
Mother's formal education (none)	Primary	-0.003	0.073
	Secondary	0.185*	0.076
	Higher	0.563***	0.117
Husband's/partner's formal education (none)	Primary	0.027	0.065
	Secondary	0.138*	0.059
	Higher	0.182*	0.077
Household wealth index quintile (poorest)	Poorer	0.024	0.084
	Middle	0.092	0.092
	Richer	0.335***	0.103
	Richest	0.494***	0.141
**Mother's Perceptions of Barrier-Free Medical Care**			
Permission (big problem)	Not a big problem	-0.040	0.076
Money (big problem)	Not a big problem	-0.029	0.065
Distance (big problem)	Not a big problem	-0.061	0.062
**Community Characteristics**			
Residential status (rural)	Urban	-0.193+	0.115
Avg. household wealth index quintile		0.080	0.077
Avg. woman's perceptions of barrier-free medical care		0.252**	0.081
Province (Punjab)	Sindh	0.574***	0.117
	Khyber P.	0.248*	0.111
	Balochistan	-0.180	0.133
	Islamabad	0.424***	0.109
Constant		-1.593***	0.220

Notes: SE=standard error. Reference categories are in parentheses. +*p*≤.10, **p*≤.05, ***p*≤.01, ****p*≤.001 (two-tailed tests).

Supplementary Table 3
Results of Heckman Probit Regression: Step 2, Outcome, Mode of Delivery, 2012-13 (*n*=3524)

		C-section (versus vaginal)	
**Variable**		**Coefficient**	**Linearized SE**
**Clinical/Medical Indications**			
Previous C-section (no)	Yes	1.950***	0.164
Past pregnancy loss (no)	Yes	0.035	0.076
Age at delivery (<20)	20-29	0.336*	0.142
	30+	0.620***	0.168
Birth order (1st)	2nd	-0.602***	0.097
	3rd	-0.535***	0.123
	4th+	-0.787***	0.130
Antenatal visits (0)	1-4	0.137	0.160
	5+	0.412+	0.215
**Type of Health Facility** (public)	Private	0.116+	0.070
Constant		-0.848**	0.285
Rho		-0.299+	
Design F(10, 434)		23.27***	

Notes: SE=standard error. Reference categories are in parentheses. +*p*≤.10, **p*≤.05, ***p*≤.01, ****p*≤.001 (two-tailed tests).

Supplementary Table 4
Results of Heckman Probit Regression: Step 1, Selection, Place of Delivery, 2017-2018 (*n*=5999)

Variable		Health facility (versus home)	
		**Coefficient**	**Linearized SE**
**Clinical/Medical Indications**			
Previous C-section (no)	Yes	1.437***	0.184
Past pregnancy loss (no)	Yes	0.015	0.056
Age at delivery (<20)	20-29	0.073	0.089
	30+	0.172+	0.099
Birth order (1st)	2nd	-0.314***	0.090
	3rd	-0.278**	0.091
	4th+	-0.516***	0.089
Antenatal visits (0)	1-4	0.717***	0.100
	5+	1.321***	0.010
**Socioeconomic Status**			
Mother's formal education (none)	Primary	0.070	0.072
	Secondary	0.123	0.096
	Higher	0.405***	0.119
Husband's/partner's formal education (none)	Primary	-0.140	0.085
	Secondary	0.024	0.068
	Higher	0.151+	0.086
Household wealth index quintile (poorest)	Poorer	0.064	0.083
	Middle	0.237*	0.106
	Richer	0.374**	0.121
	Richest	0.578***	0.160
**Mother's Perceptions of Barrier-Free Medical Care**			
Permission (big problem)	Not a big problem	-0.035	0.087
Money (big problem)	Not a big problem	0.212***	0.063
Distance (big problem)	Not a big problem	-0.014	0.061
**Community Characteristics**			
Residential status (rural)	Urban	-0.008	0.097
Avg. household wealth index quintile		0.063	0.057
Avg. woman's perceptions of barrier-free medical care		-0.106	0.100
Province (Punjab)	Sindh	0.395**	0.130
	Khyber P.	-0.019	0.125
	Balochistan	-0.472**	0.150
	Islamabad	0.081	0.125
Constant		-0.481*	0.222

Notes: SE=standard error. Reference categories are in parentheses. +*p*≤.10, **p*≤.05, ***p*≤.01, ****p*≤.001 (two-tailed tests).

Supplementary Table 5
Results of Heckman Probit Regression: Step 2, Outcome, Mode of Delivery, 2012-13 (*n*=4029)

		C-section (versus vaginal)	
**Variable**		**Coefficient**	**Linearized SE**
**Clinical/Medical Indications**			
Previous C-section (no)	Yes	1.987***	0.146
Past pregnancy loss (no)	Yes	0.152*	0.066
Age at delivery (<20)	20-29	0.406***	0.113
	30+	0.638***	0.140
Birth order (1st)	2nd	-0.472***	0.102
	3rd	-0.440***	0.110
	4th+	-0.564***	0.133
Antenatal visits (0)	1-4	-0.151	0.240
	5+	-0.036	0.306
**Type of Health Facility** (public)	Private	0.434***	0.065
Constant		-0.668+	0.343
Rho		-0.675***	
Design F(10, 408)		28.51***	

Notes: SE=standard error. Reference categories are in parentheses. +*p*≤.10, **p*≤.05, ***p*≤.01, ****p*≤.001 (two-tailed tests).

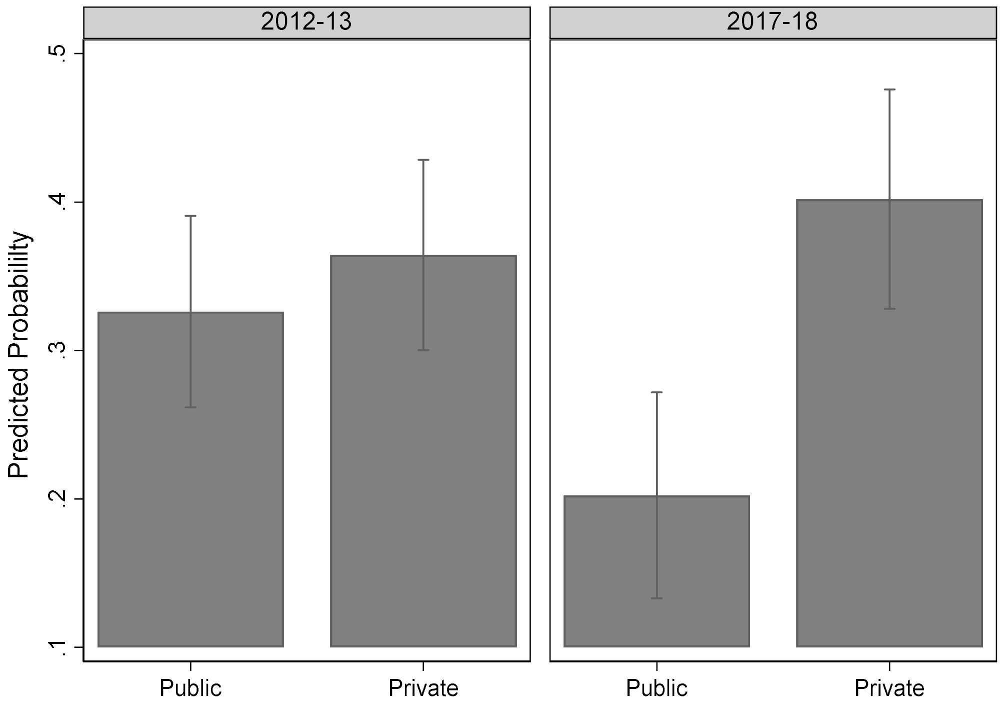

Supplementary Fig. 1 Effect of type of health facility on predicted probability of C-section delivery over time. *n* = 9083. Respondents from Khyber Pakhtunkhwa excluded.
